# Systematic review and meta-analysis of vaginal natural orifice transluminal endoscopic surgery vs laparoscopic hysterectomy

**DOI:** 10.1016/j.xagr.2024.100320

**Published:** 2024-02-08

**Authors:** Greg J. Marchand, Ahmed Taher Masoud, Hollie Ulibarri, Amanda Arroyo, Carmen Moir, Madison Blanco, Daniela Gonzalez Herrera, Brooke Hamilton, Kate Ruffley, Mary Petersen, Sarena Fernandez, Ali Azadi

**Affiliations:** a Marchand Institute for Minimally Invasive Surgery (Drs Marchand and Masoud and Mses Ulibarri, Arroyo, Moir, Blanco, Gonzalez Herrera, Hamilton, and Ruffley), Mesa, AZ; b Faculty of Medicine, Fayoum University (Dr Masoud), Fayoum, Egypt; c Midwestern University College of Osteopathic Medicine (Mses Petersen and Fernandez), Glendale, AZ; d College of Medicine, University of Arizona (Dr Azadi), Phoenix, AZ; e School of Medicine, Creighton University (Dr Azadi), Phoenix, AZ

**Keywords:** hysterectomy, laparoscopy, vNOTES

## Abstract

**OBJECTIVE:**

Because vaginal natural orifice transluminal endoscopic surgery and laparoscopic hysterectomy techniques both aim to decrease tissue injury and postoperative morbidity and mortality and to improve a patient's quality of life, we sought to evaluate the safety and effectiveness of a hysterectomy by vaginal natural orifice transluminal endoscopic surgery and compared that with conventional laparoscopic hysterectomy among women with benign gynecologic diseases.

**DATA SOURCES:**

We used Scopus, Medline, ClinicalTrials.Gov, PubMed, and the Cochrane Library and searched from database inception to September 1, 2023.

**STUDY ELIGIBILITY CRITERIA:**

We included all eligible articles that compared vaginal natural orifice transluminal endoscopic surgery hysterectomy with any conventional laparoscopic hysterectomy technique without robotic assistance for women with benign gynecologic pathology and that included at least 1 of our main outcomes. These outcomes included estimated blood loss (in mL), operation time (in minutes), length of hospital stay (in days), decrease in hemoglobin level (g/dL), visual analog scale pain score on postoperative day 1, opioid analgesic dose required, rate of conversion to another surgical technique, intraoperative complications, postoperative complications, and requirements for blood transfusion. We included randomized controlled trials and observational studies. Ultimately, 14 studies met our criteria.

**METHODS:**

The study quality of the randomized controlled trials was assessed using the Cochrane assessment tool, and the quality of the observational studies was assessed using the ROBINS-I tool. We analyzed data using RevMan 5.4.1. Continuous outcomes were analyzed using the mean difference and 95% confidence intervals under the inverse variance analysis method. Dichotomous outcomes were analyzed using OpenMeta[Analyst] and odds ratios and 95% confidence intervals were reported.

**RESULTS:**

The operative time and length of hospitalization were shorter in the vaginal natural orifice transluminal endoscopic surgery cohort. We also found lower visual analog scale pain scores, fewer postoperative complications, and fewer blood transfusions in the vaginal natural orifice transluminal endoscopic surgery group. We found no difference in the estimated blood loss, decrease in hemoglobin levels, analgesic usage, conversion rates, or intraoperative complications.

**CONCLUSION:**

When evaluating the latest data, it seems that vaginal natural orifice transluminal endoscopic surgery techniques may have some advantages over conventional laparoscopic hysterectomy techniques.

**AJOG Global Reports at a Glance**

**Why was this study conducted?**

The best technique for minimally invasive hysterectomy for benign disease is widely debated and extremely controversial and therefore we wanted to compare vaginal natural orifice transluminal endoscopic surgery (vNOTES) with laparoscopic hysterectomy in terms of safety and efficacy.

**Key findings**

The vNOTES techniques had a shorter operative time, a shorter hospital stay, and fewer postoperative complications.

**What does this add to what is known?**

This study adds to the evidence that a vNOTES hysterectomy may be superior to a laparoscopic hysterectomy in some attributes and that both are valid techniques.

## Introduction

Hysterectomy is considered one of the most common gynecologic surgeries among women in the United States,^1^^,^^2^ and benign gynecologic diseases are responsible for about 90% of the hysterectomies.^3^ In a recent Cochrane review^3^ it was reported that a hysterectomy via the vaginal route may be the preferred route when considering the efficacy and safety of the different surgical techniques. Although hysterectomy via the vaginal route has been associated with shorter hospitalization, fewer infections, and earlier return to the normal daily activity, it may be limited by the poor visualization, difficult manipulation, and the lack of training and skills among surgeons.^3^, ^4^, ^5^

Minimally invasive procedures, such as laparoscopy, natural orifice transluminal endoscopic surgery (NOTES), robotic assisted laparoscopy, and single port laparoscopic surgery aim to decrease tissue injury and postoperative morbidity and mortality and to improve the patient's quality of life.^6^, ^7^, ^8^ In NOTES, surgeons use natural orifices in the human body to reach the abdominal cavity and perform surgeries. This has the potential to improve the recovery and cosmesis of surgery when compared with the standard laparotomy and laparoscopic techniques that use new incisions in the abdominal wall as access points.^9^, ^10^, ^11^ Vaginal NOTES (vNOTES) can be used for many different procedures in the female abdomen and pelvis, including surgeries on the uterus, ovaries, and gastrointestinal tract.^12^ Recently, there has been increasing interest in hysterectomy by vNOTES. This was first described by Su et al^13^ in 2012, and the technique may combine many of the advantages of vaginal and laparoscopic techniques.^14^^,^^15^ Since that time, other published studies have investigated hysterectomy by the vNOTES technique in terms of operative time, postoperative pain, intraoperative and postoperative complication rates, duration of hospitalization, and cost.^16^, ^17^, ^18^, ^19^ Many of these studies have demonstrated that vNOTES may be a valid and reliable hysterectomy option for minimally invasive hysterectomy.^20^^,^^21^

### Objective

Building on the previous studies on this topic, we set out to perform a systematic review and meta-analysis to evaluate the safety and effectiveness of hysterectomy by vNOTES in comparison with a conventional laparoscopic hysterectomy in women with benign gynecologic diseases.

## Materials and Methods

We used the Preferred Reporting Items for Systematic Reviews and Meta-Analyses statement as a guideline to conduct this systematic review and meta-analysis.^22^

### Search and information databases

We used the following search strategy in our search from inception of each database to September 1, 2023: (“Hysterectomy” OR hysterectom*) AND ((VANH OR VAMIS OR TVNH OR “glove” AND “port”) OR gloveport OR “single port” OR “single incision laparoscopic surgery” OR SILS OR “laparo-endoscopic single site” OR “laparoendoscopic single site”) OR (“Natural Orifice Endoscopic Surgery” OR NOTES OR vNOTES OR (“natural” AND “orifice” AND “endoscop*”))). Scopus, Medline, ClinicalTrials.Gov, PubMed, and the Cochrane Library were the used online databases.

### Selection criteria and eligibility criteria

We selected eligible studies in 2 steps. We first screened titles and abstracts to select relevant studies that were further evaluated to reach the final eligible articles based on our inclusion criteria. We included studies that compared hysterectomy by vNOTES with hysterectomy by any conventional laparoscopy technique without robotic assistance among women with benign gynecologic disorders. Single-arm studies, articles that did not evaluate any of our outcomes, and secondary research, such as systematic reviews and meta-analyses, were excluded.

### Data extraction

Data from eligible articles was extracted manually. We extracted the general information of the studies, in addition to the demographic data of included patients, including age, body mass index, parity, the number of previous surgeries, uterine weight, the number of previous cesarean deliveries, and the indication for surgery. We extracted data on the primary outcomes, including estimated blood loss (mL), operation time (minutes), length of hospital stay (days), decrease in hemoglobin level (g/dL), visual analog scale (VAS) pain score at day 1, the required analgesic dose, the rate of conversion to another surgical technique, the rate of intraoperative complications, the rate of postoperative complications, and the rate of requiring blood transfusion. Finally, we retrieved all of the required data for the risk of bias assessment.

### Quality assessment

We included both observational studies and randomized controlled trials (RCTs). We used the Cochrane assessment tool to assess the quality of the clinical trials.^23^ RCTs were categorized as high, moderate, or low quality based on the state of randomization, allocation concealment, sequence generation, adequate blinding, if the missing outcome data were adequately addressed, and if the trial was free of selective reporting. Regarding the quality assessment of included observational studies, we used the ROBINS-I tool.^24^

### Statistical analysis

We analyzed data of our continuous outcomes using RevMan 5.4.1. Continuous outcomes were analyzed using mean difference (MD) and 95% confidence intervals (CIs) under the inverse variance analysis method. Dichotomous outcomes were analyzed with the help of OpenMeta[Analyst]^25^ using odds ratios (OR) and 95% CIs. We conducted subgroup analyses according to the study design of the included studies. The heterogeneity among the studies was assessed using the *P* value of the chi-square test and the I^2^ statistic. The outcome was considered heterogeneous if *P*<.1 or if I^2^>50%. In all cases, we tried to solve the inconsistency in data using subgroup analyses.^26^

## Results

### Search results and summary of the included studies

**Figure 1:**
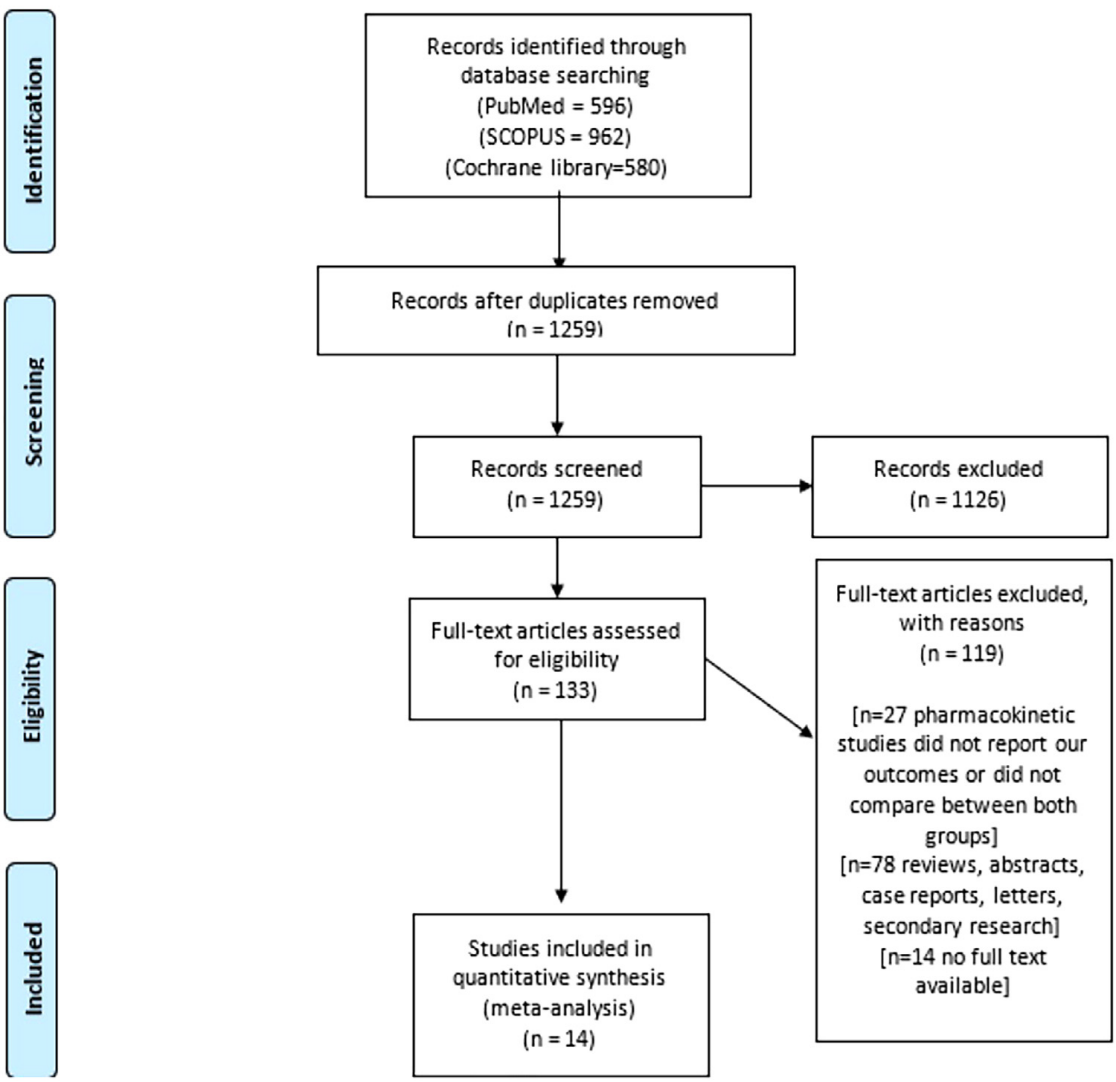
PRISMA flow diagram *PRISMA*, Preferred Reporting Items for Systematic Reviews and Meta-Analyses.

The results of our electronic search are summarized in the Preferred Reporting Items for Systematic Reviews and Meta-Analyses flow diagram (Figure 1). We included 14 eligible studies in our meta-analysis^15^^,^^27^, ^28^, ^29^, ^30^, ^31^, ^32^, ^33^, ^34^, ^35^, ^36^, ^37^, ^38^, ^39^ with a total of 1310 patients who underwent a hysterectomy for benign disease. A total of 539 patients underwent hysterectomy by vNOTES, whereas 771 patients underwent hysterectomy by conventional laparoscopic techniques. The mean age of the included participants was 47.5 years. Table 1^15^^,^^27^, ^28^, ^29^, ^30^, ^31^, ^32^, ^33^, ^34^, ^35^, ^36^, ^37^, ^38^, ^39^ shows the baseline characteristics of the included patients. Table 2 ^15^^,^^27^, ^28^, ^29^, ^30^, ^31^, ^32^, ^33^, ^34^, ^35^, ^36^, ^37^, ^38^, ^39^ shows the indications for surgery for those patients.

Marchand. Vaginal natural orifice transluminal endoscopic surgery vs laparoscopic hysterectomy. Am J Obstet Gynecol Glob Rep 2024.

**Table 1:** Baseline characteristics of the included studies

Study	Study design	Sample size		Age (y) (mean ± SD)		BMI (kg/m^2^)		Parity (mean ± SD)		Previous surgery, n (%)		Uterine weight (g)		Previous cesarean delivery, n (%)	
		vNOTES	Laparoscopy	vNOTES	Laparoscopy	vNOTES	Laparoscopy	vNOTES	Laparoscopy	vNOTES	Laparoscopy	vNOTES	Laparoscopy	vNOTES	Laparoscopy
Badiglian-Filho et al,^27^ 2021	Retrospective cohort	21	65	47.19±11.11	46.69±9.11	27.19±4.42	28.32±5.78	NR	NR	6 (28.6)	16 (24.6)	129.12±88.02	126.15±87.96	12 (57.2)	41 (63.1)
Baekelandt et al,^15^ 2019	RCT	35	35	45.25±9.7	50±8.8	29±6.1	28.5±5.7	NR	NR	20 (57)	16 (46)	311±176.8	243±133	8 (23)	5 (14)
Baron et al,^28^ 2022	Retrospective study	36	50	46.5±6.6	49.25±9.5	28±5.2	26.5±4.45	NR	NR	NR	NR	493.5±331	582.25±361.7	4 (11)	18 (36)
Basol et al,^29^ 2021	Retrospective study	20	40	49.85±5.25	49.10±5.77	26.97±4.37	27.07±3.71	2.85±1.09	2.9±1.4	5 (25)	17 (42.5)	NR	NR	NR	NR
Kaya et al,^30^ 2020	Cross-sectional study	30	30	51.58±7.31	50.71±7.33	31.41±8.16	30.76±6.43	2.57±1.68	2.51±1.13	10 (32.5)	29 (42.6)	NR	NR	NR	NR
Kaya et al,^31^ 2022	Cross-sectional study	48	35	54.5±7.6	52.25±7.3	37.1±5.5	33.85±2.9	3.75±2	3±1.9	1 (2)	0	343.75±160.4	239.75±86.7	NR	NR
Kaya et al,^32^ 2022	Cross-sectional study	30	48	47.5±3.67	51.5±8.5	31.4±6.5	32.6±5.9	3±1.47	2.5±1.34	0	0	456.25±131.1	452.5±114.4	NR	NR
Kim et al,^33^ 2018	Retrospective study	40	120	47.3±6.1	46.4±4.7	24.3±2.6	23.5±3.4	1.75±.7	2±0.78	30 (75)	76 (63.3)	278.3±168.9	287.2±127.4	NR	NR
Noh et al,^34^ 2022	Prospective cohort study	33	40	48.0±4.1	47.5±4.7	22.5±.74	23.72±1.08	NR	NR	7 (21.21)	9 (22.5)	317.9±161.1	408.8±252.3	NR	NR
Park et al,^35^ 2021	RCT	13	13	55±12.55	48.25±8.6	24.13±1.87	22.4±1.7	NR	NR	NR	NR	364.2±268.7	207.75±75.61	4 (30.8)	3 (23)
Puisungnoen et al,^36^ 2020	Retrospective study	50	50	47.3±6.7	48.2±5.8	24.7±4.4	24.5±4.2	NR	NR	18 (36.0)	20 (40.0)	159	231.5	NR	NR
Wang et al,^37^ 2015	Retrospective study	147	147	46.1±4.7	45.9±4.7	24.5±3.8	24.7±3.9	NR	NR	NR	NR	397.2±182.3	480.2±305.9	NR	NR
Yang et al,^39^ 2014	Retrospective study	16	32	47.3±4.6	45.8±5.4	23.8±2.3	23.9±3.7	1.75±0.84	2±0.96	7 (43.8)	11 (34.4)	299.4±186	292.7±136	NR	NR
Yang et al,^38^ 2020	Retrospective study	20	66	46.5±4.40	45.8±3.97	22.5±2.36	22.6±2.52	NR	NR	NR	NR	219.9±148.4	257.6±169.5	NR	NR

*NR*, not reported; *RCT*, randomized clinical trial; *vNOTES*, transvaginal natural orifice transluminal endoscopic surgery.

**Table 2:** Indications for surgery in the included studies

Study	Indication for surgery, n (%)									
	Myoma		Endometrial hyperplasia		Bleeding		Adnexal mass		Adenomyosis	
	vNOTES	Laparoscopy	vNOTES	Laparoscopy	vNOTES	Laparoscopy	vNOTES	Laparoscopy	vNOTES	Laparoscopy
Badiglian-Filho et al,^27^ 2021	NR	NR	1 (4.8)	6 (9.2)	NR	NR	NR	NR	NR	NR
Baekelandt et al,^15^ 2019	17 (49)	16 (45)	2 (6)	2 (6)	5 (14)	2 (6)	NR	NR	6 (17)	6 (17)
Baron et al,^28^ 2022	14 (39)	20 (40)	NR	NR	4 (11.1)	6 (12)	NR	NR	3^3^^,^^8^	17 (34)
Basol et al,^29^ 2021	NR	NR	NR	NR	10 (50)	32 (80)	2 (10)	2 (5)	NR	NR
Kaya et al,^30^ 2020	21 (74.2)	44 (63.2)	1 (3.2)	0	3 (9.6)	9 (13.2)	4 (12.9)	10 (14.7)	10 (32.3)	23 (33.8)
Kaya et al,^31^ 2022	18 (37.5)	13 (37.1)	11 (23)	7 (20)	16 (33.1)	13 (37.1)	3 (6.3)	2 (8.6)	16 (33.3)	12 (34.4)
Kaya et al,^32^ 2022	25 (83.3)	39 (81.3)	1 (3.3)	0	14 (64.7)	23 (47.9)	NR	NR	4 (13.4)	9 (18.8)
Kim et al,^33^ 2018	12 (30)	37 (30.8)	NR	NR	NR	NR	NR	NR	10 (25)	36 (30)
Noh et al,^34^ 2022	23 (69.7)	26 (65.0)	NR	NR	NR	NR	NR	NR	5 (15.2)	11 (27.5)
Park et al,^35^ 2021	7 (53.8)	8 (61.5)	1 (7.7)	2 (15.4)	0	1 (7.7)	NR	NR	2 (15.4)	0
Puisungnoen et al,^36^ 2020	24 (48.0)	23 (46.0)	NR	NR	NR	NR	NR	NR	19 (38.0)	20 (40.0)
Wang et al,^37^ 2015	NR	NR	NR	NR	NR	NR	NR	NR	NR	NR
Yang et al,^39^ 2014	NR	NR	NR	NR	NR	NR	NR	NR	NR	NR
Yang et al,^38^ 2020	5 (25)	34 (51.5)	2 (10)	1 (1.52)	13 (65)	31 (47)	NR	NR	9 (45)	26 (39.4)

*NR*, not reported; *vNOTES*, transvaginal natural orifice transluminal endoscopic surgery.

### Results of the quality assessment

**Figure 2:**
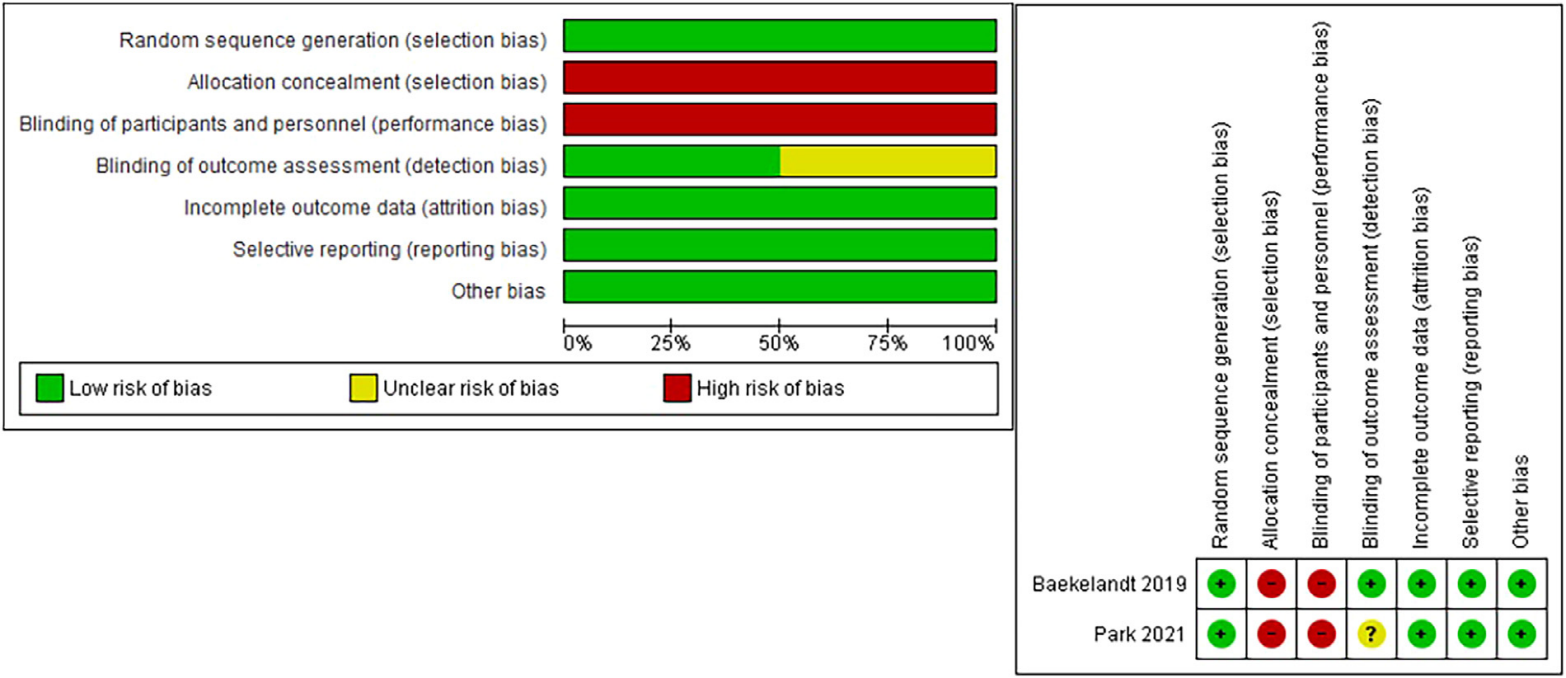
Results of the Cochrane tool risk of bias assessment of the RCTs *RCTs*, randomized controlled trials.

To assess the risk of bias in the included clinical trials, we used the Cochrane Risk of Bias (ROB) tool^40^ (Figure 2), and the remaining 12 included observational studies were evaluated using the ROBINS-I tool.^24^ The retrospective nature of the observational studies yielded moderate bias in the measurement of outcomes domain, selection domain, and publication bias. A detailed illustration of the quality assessment of observational studies is shown in Table 3.

*Marchand. Vaginal natural orifice transluminal endoscopic surgery vs laparoscopic hysterectomy. Am J Obstet Gynecol Glob Rep 2024.*

**Table 3:** Risk of bias of the included observational studies

Study	Bias owing to confounding	Selection bias	Bias in classification of interventions	Bias owing to deviations from intended intervention	Bias owing to missing data	Bias in measurement of outcomes	Bias in selection of reported result
Badiglian-Filho et al,^27^ 2021	Moderate	Moderate	Low	Low	Low	Moderate	Low
Baron et al,^28^ 2022	Moderate	Moderate	Low	Low	Low	Moderate	Low
Basol et al,^29^ 2021	Moderate	Moderate	Low	Low	Low	Moderate	Low
Kaya et al,^30^ 2020	Moderate	Moderate	Low	Low	Low	Moderate	Low
Kaya et al,^31^ 2022	Moderate	Moderate	Low	Low	Low	Moderate	Moderate
Kaya et al,^32^ 2022	Moderate	Moderate	Low	Low	Low	Moderate	Moderate
Kim et al,^33^ 2018	Moderate	Moderate	Low	Low	Low	Moderate	Low
Noh et al,^34^ 2022	Moderate	Moderate	Low	Low	Low	Moderate	Low
Puisungnoen et al,^36^ 2020	Moderate	Moderate	Low	Low	Low	Moderate	Low
Wang et al,^37^ 2015	Moderate	Moderate	Low	Low	Low	Low	Low
Yang et al,^39^ 2014	Moderate	Moderate	Low	Low	Low	Moderate	Low
Yang et al,^38^ 2020	Moderate	Moderate	Low	Low	Low	Moderate	Low

### Analysis of outcomes

#### Operation time (in minutes)

**Figure 3:**
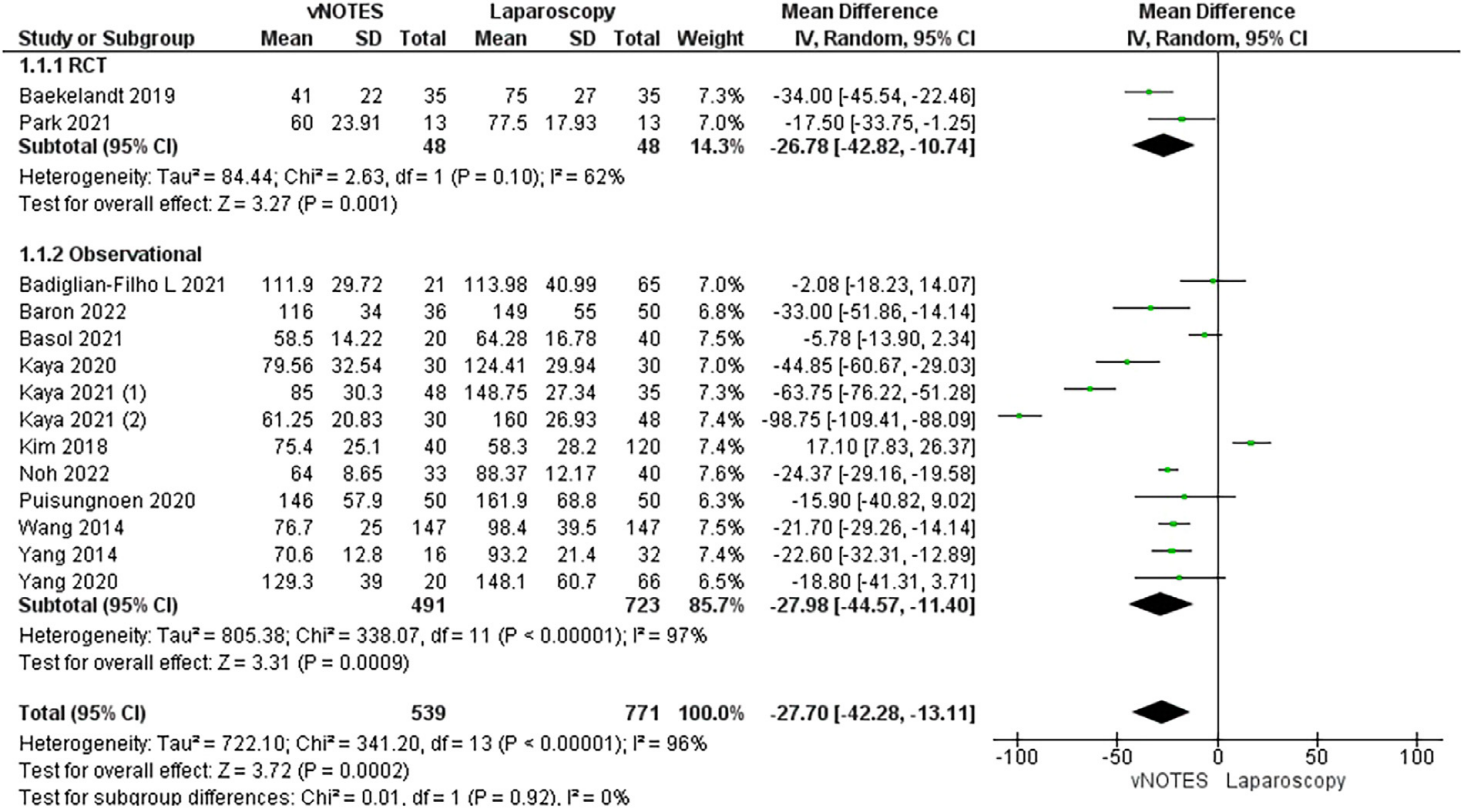
Meta-analysis of the total operation time (in minutes) *CI*, confidence interval; *IV*, inverse variance; *RCT*, randomized controlled trial; *SD*, standard deviation; *vNOTES*, vaginal natural orifice transluminal endoscopic surgery.

All included studies assessed the operation time.^15^^,^^27^^,^^29^, ^30^, ^31^, ^32^, ^33^, ^34^, ^35^, ^36^, ^37^, ^38^, ^39^ We conducted a subgroup analysis according to the study design of the included studies. The RCT subgroup included 2 RCTs.^15^^,^^35^ Our research showed that a hysterectomy by vNOTES surgical techniques was favored over the conventional laparoscopic techniques (MD, −26.78; −42.82 to −10.74; *P*=.001). The observational subgroup included 12 observational studies.^27^, ^28^, ^29^, ^30^, ^31^, ^32^, ^33^, ^34^^,^^36^, ^37^, ^38^, ^39^ We observed that the operation time was much shorter with the vNOTES technique than with the laparoscopic techniques (MD, −27.98; −44.57 to −11.40; *P*=.001). The overall analysis favored the vNOTES surgical technique (MD, −27.70; −42.28 to −13.11; *P*=.002). The pooled analysis was heterogeneous (*P*<.01; I²=97%) (Figure 3).

*Marchand. Vaginal natural orifice transluminal endoscopic surgery vs laparoscopic hysterectomy. Am J Obstet Gynecol Glob Rep 2024.*

#### Estimated blood loss (in mL)

**Figure 4:**
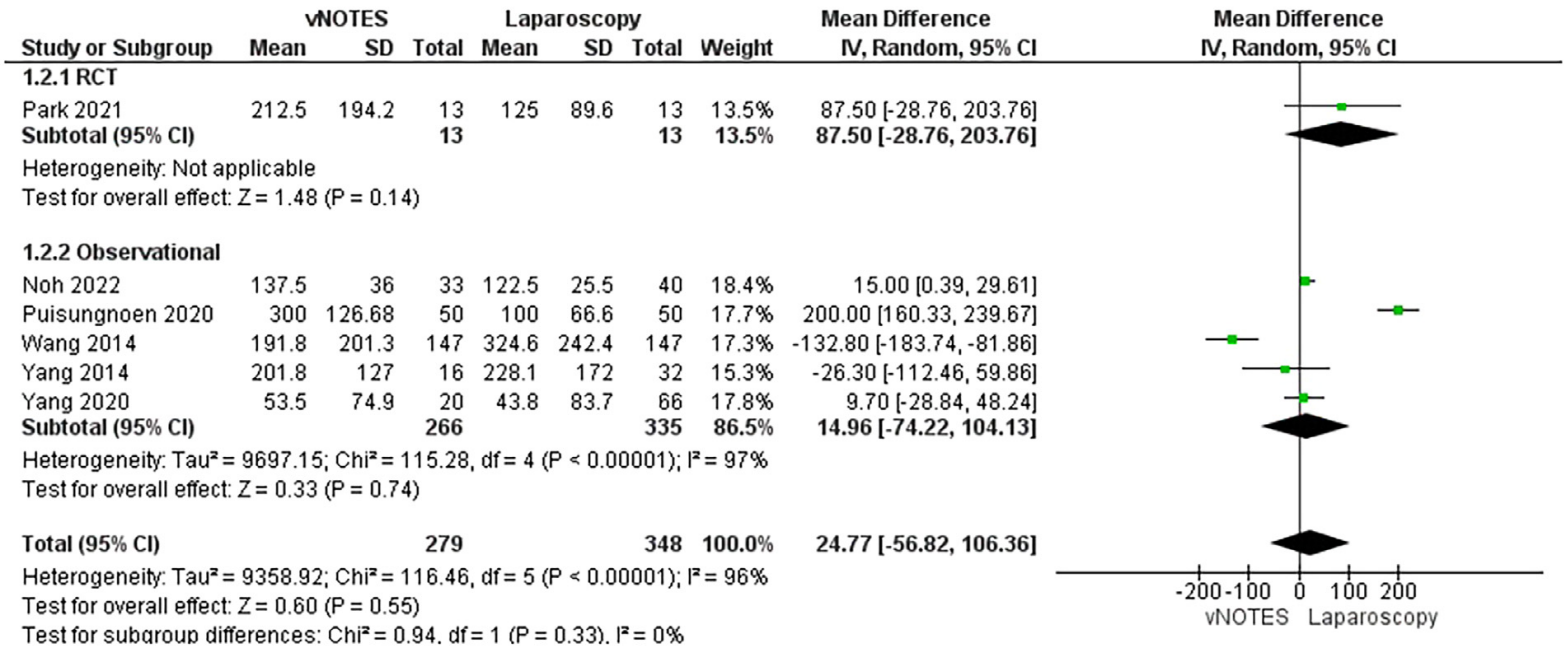
Meta-analysis of estimated blood loss (in mL) *CI*, confidence interval; *IV*, inverse variance; *RCT*, randomized controlled trial; *SD*, standard deviation; *vNOTES*, vaginal natural orifice transluminal endoscopic surgery.

Six studies reported estimated blood loss.^34^, ^35^, ^36^, ^37^, ^38^, ^39^ The estimated blood loss was similar in both surgical techniques (MD, 24.77; −56.82 to 106.36; *P*=.55). The data were heterogeneous (*P*<.001; I²=96%) (Figure 4).

*Marchand. Vaginal natural orifice transluminal endoscopic surgery vs laparoscopic hysterectomy. Am J Obstet Gynecol Glob Rep 2024.*

#### Length of hospital stay (in days)

**Figure 5:**
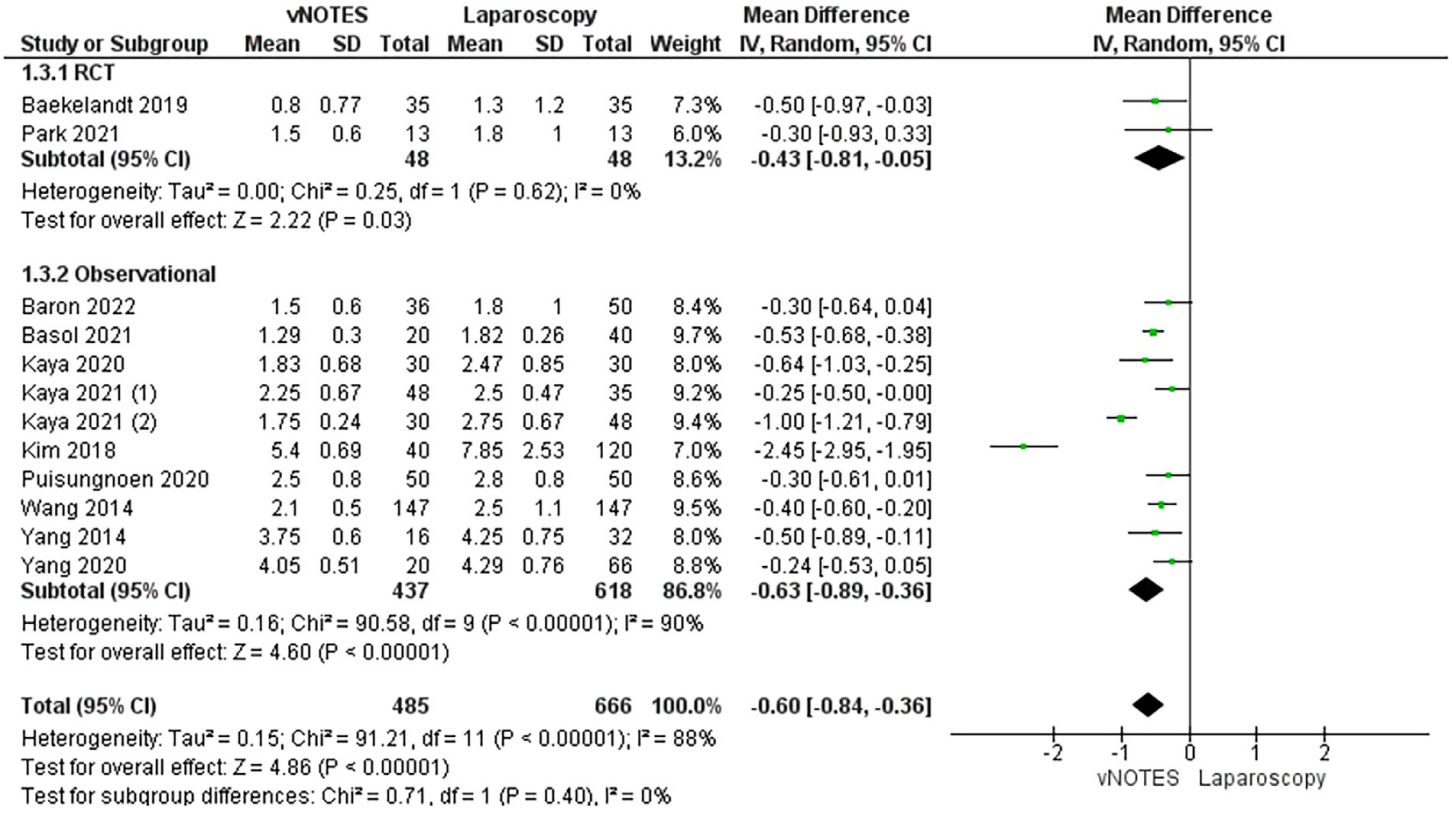
Meta-analysis of length of hospital stay (in days) *CI*, confidence interval; *IV*, inverse variance; *RCT*, randomized controlled trial; *SD*, standard deviation; *vNOTES*, vaginal natural orifice transluminal endoscopic surgery.

The duration of hospitalization was assessed in 12 studies.^15^^,^^28^, ^29^, ^30^, ^31^, ^32^, ^33^^,^^35^, ^36^, ^37^, ^38^, ^39^ The combined MD showed a significantly shorter period of hospitalization among patients who underwent hysterectomy by vNOTES techniques than among those who underwent hysterectomy by conventional laparoscopic techniques (MD, −0.60; −0.84 to −0.36; *P*<.001). Heterogeneity was present in the analyzed data (*P*<.001; I²=88%) (Figure 5).

*Marchand. Vaginal natural orifice transluminal endoscopic surgery vs laparoscopic hysterectomy. Am J Obstet Gynecol Glob Rep 2024.*

#### Decrease in hemoglobin levels (g/dL)

**Figure 6:**
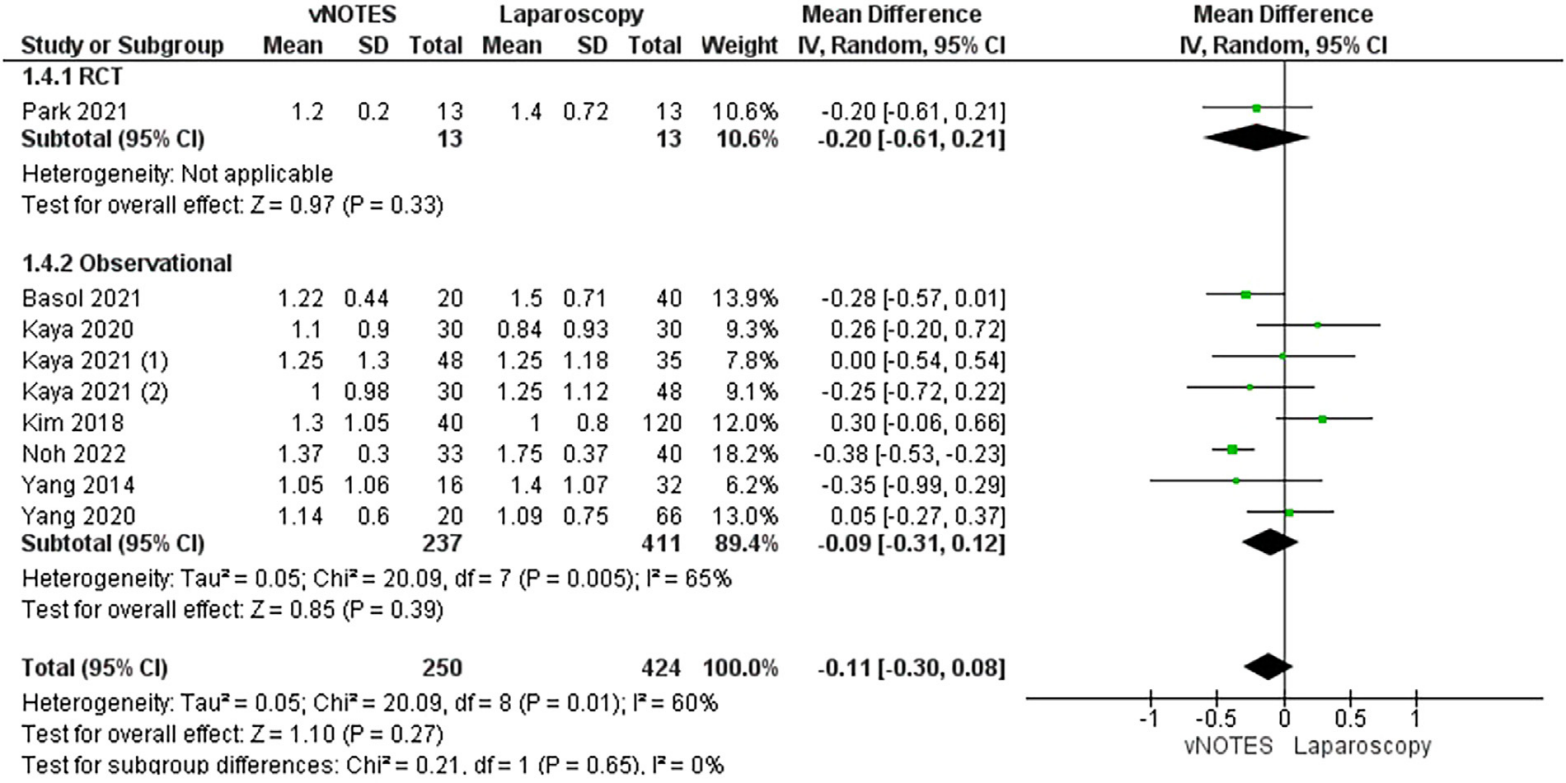
Meta-analysis of the decrease in hemoglobin levels (g/dL) *CI*, confidence interval; *IV*, inverse variance; *RCT*, randomized controlled trial; *SD*, standard deviation; *vNOTES*, vaginal natural orifice transluminal endoscopic surgery.

This outcome was reported in 9 studies.^29^, ^30^, ^31^, ^32^, ^33^, ^34^, ^35^^,^^38^^,^^39^ Our analysis revealed a comparable decrease in the hemoglobin level in each group (MD, −0.11; −0.30 to 0.08; *P*=.27). We observed moderate heterogeneity in the data (*P*=.01; I²=60%) (Figure 6).

*Marchand. Vaginal natural orifice transluminal endoscopic surgery vs laparoscopic hysterectomy. Am J Obstet Gynecol Glob Rep 2024.*

#### Visual analog scale score on postoperative day 1

**Figure 7:**
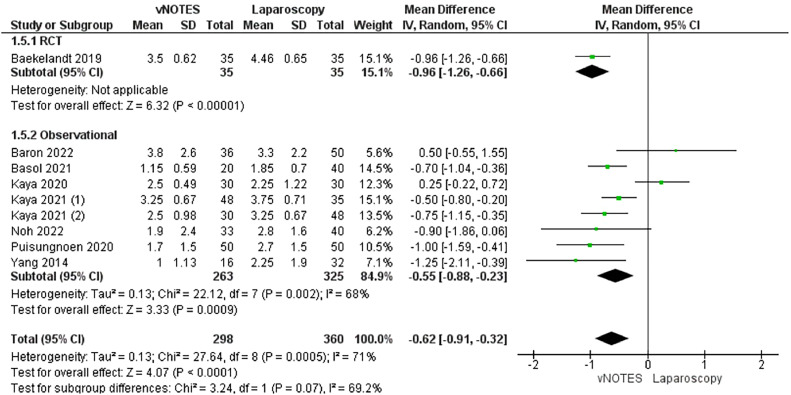
Meta-analysis of the VAS score on postoperative day 1 *CI*, confidence interval; *IV*, inverse variance; *RCT*, randomized controlled trial; *SD*, standard deviation; *VAS*, visual analog scale; *vNOTES*, vaginal natural orifice transluminal endoscopic surgery.

Nine studies evaluated the VAS score of included patients on postoperative day 1.^15^^,^^28^, ^29^, ^30^, ^31^, ^32^^,^^34^^,^^36^^,^^39^ Hysterectomy by vNOTES was associated with significantly lower VAS scores on the first day after operation (MD, −0.62; −0.91 to −0.32; *P*<.001). We found substantial heterogeneity among the data (*P*=.05; I²=71%) (Figure 7).

*Marchand. Vaginal natural orifice transluminal endoscopic surgery vs laparoscopic hysterectomy. Am J Obstet Gynecol Glob Rep 2024.*

#### Required postoperative analgesic dose

**Figure 8:**
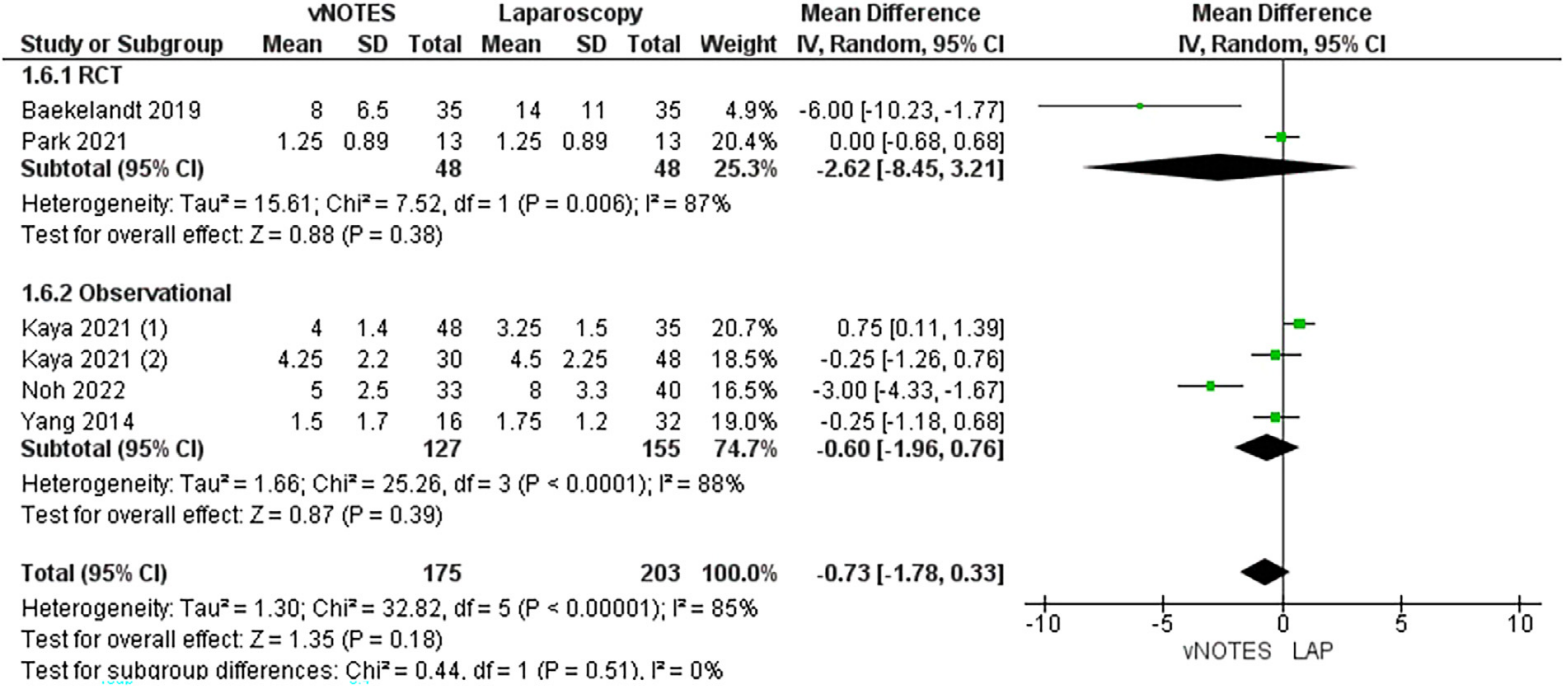
Meta-analysis of the required postoperative analgesia dose *CI*, confidence interval; *IV*, inverse variance; *RCT*, randomized controlled trial; *SD*, standard deviation; *vNOTES*, vaginal natural orifice transluminal endoscopic surgery.

The required analgesic doses were measured by 6 studies.^15^^,^^31^^,^^32^^,^^34^^,^^35^^,^^39^ We found no significant variation between the surgical techniques in terms of the required analgesic doses (MD, −0.73; −1.78 to 0.33) (*P*=.18). Data were heterogeneous (*P*<.001; I²=85%) (Figure 8).

*Marchand. Vaginal natural orifice transluminal endoscopic surgery vs laparoscopic hysterectomy. Am J Obstet Gynecol Glob Rep 2024.*

#### Conversion to another surgical technique

**Figure 9:**
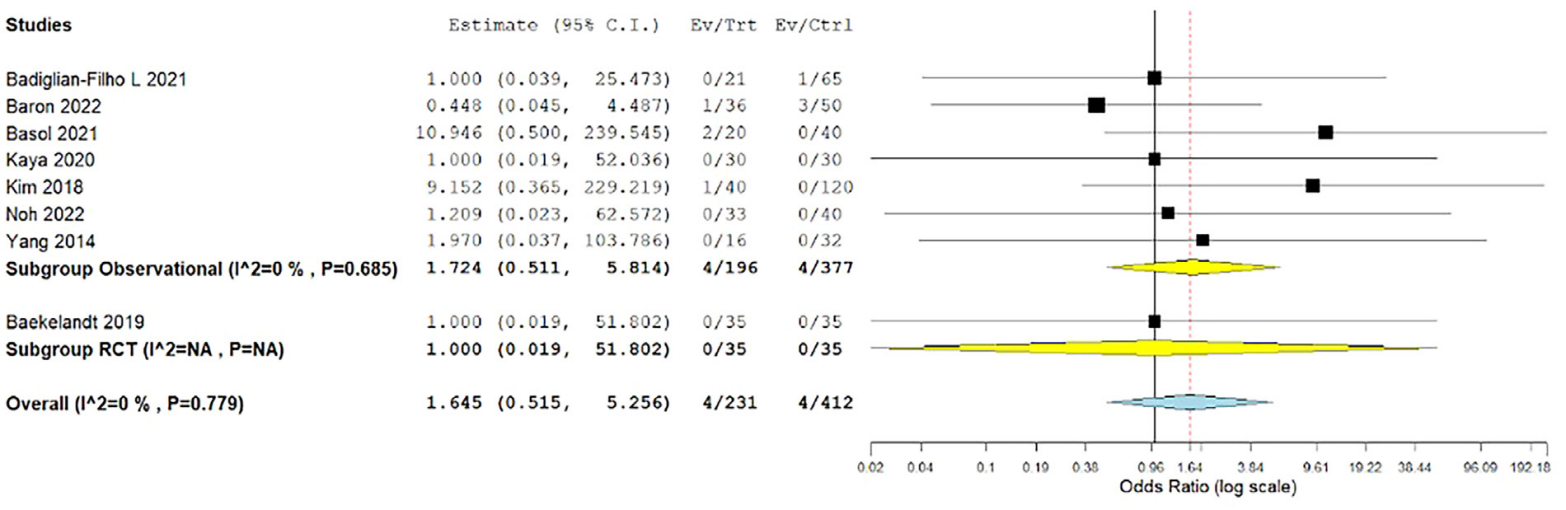
Meta-analysis of the rate of conversion to another surgical technique *CI*, confidence interval; *RCT*, randomized controlled trial.

Eight studies reported the conversion rate to another surgical technique.^15^^,^^27^, ^28^, ^29^, ^30^^,^^33^^,^^34^^,^^39^ The overall OR showed no significant difference between the groups (OR, 1.645; 0.515–5.256; *P*=.401). The combined analysis was homogenous (*P*=.779; I²=0%) (Figure 9).

*Marchand. Vaginal natural orifice transluminal endoscopic surgery vs laparoscopic hysterectomy. Am J Obstet Gynecol Glob Rep 2024.*

#### Intraoperative complications

**Figure 10:**
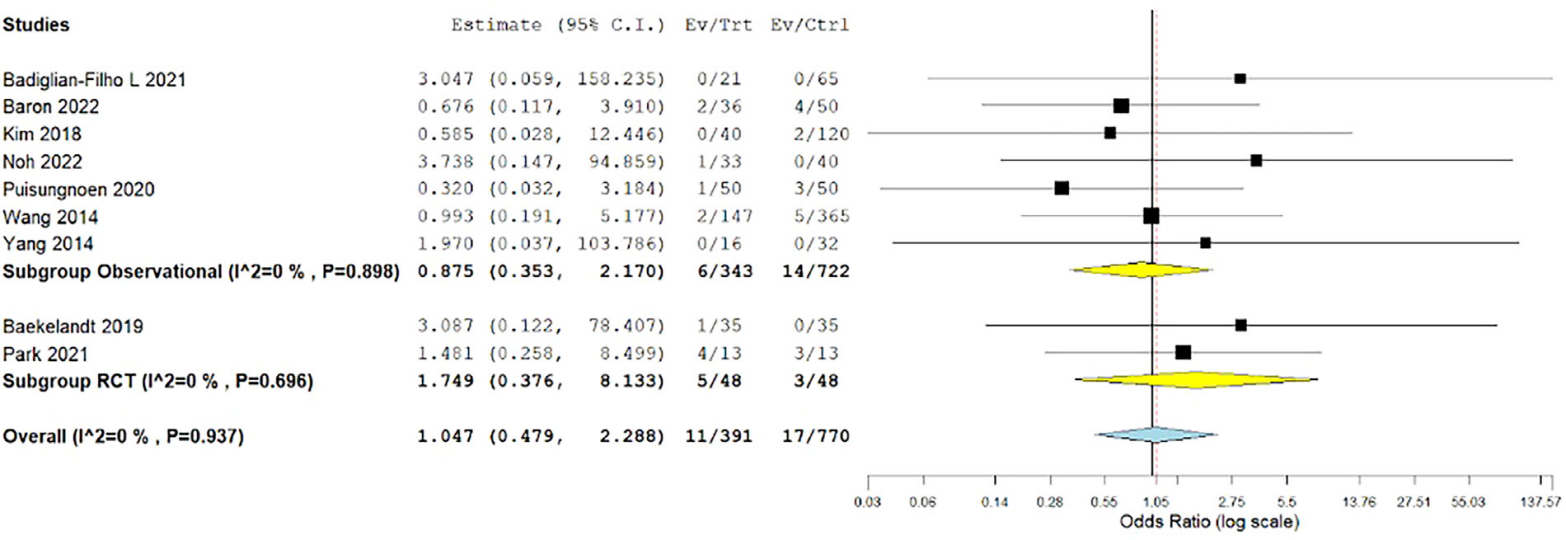
Meta-analysis of the rate of intraoperative complications *CI*, confidence interval; *RCT*, randomized controlled trial.

Intraoperative complications were evaluated by 9 studies.^15^^,^^27^^,^^28^^,^^33^, ^34^, ^35^, ^36^, ^37^^,^^39^ Our analysis demonstrated that both surgical techniques showed similar intraoperative complication rates (OR, 1.047; 0.479–2.288; *P*=.909). The overall analysis was homogenous (*P*=.937; I²=0%) (Figure 10).

*Marchand. Vaginal natural orifice transluminal endoscopic surgery vs laparoscopic hysterectomy. Am J Obstet Gynecol Glob Rep 2024.*

#### Postoperative complications

**Figure 11:**
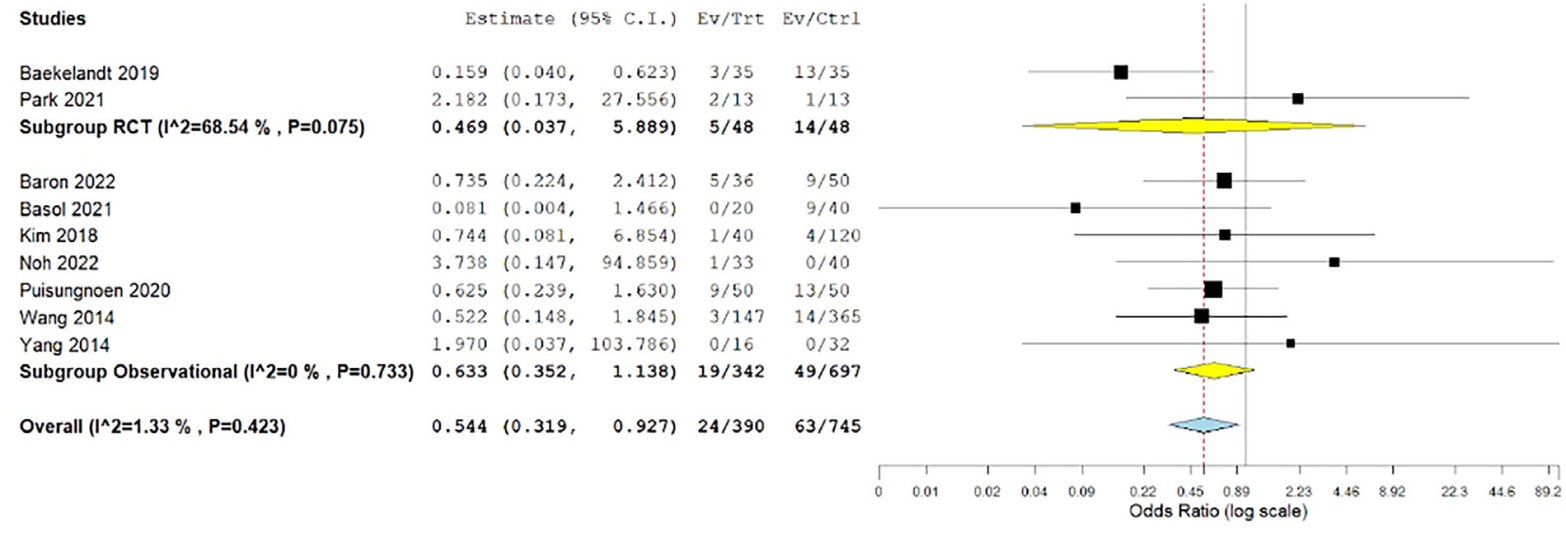
Meta-analysis of the rate of postoperative complications *CI*, confidence interval; *RCT*, randomized controlled trial.

The overall odds ratio showed a significantly lower incidence of postoperative complications among patients who underwent hysterectomy by the vNOTES technique (OR, 0.544; 0.319–0.927; *P*=.025). The data were homogeneous (*P*=.423; I²=1.33%) (Figure 11).

*Marchand. Vaginal natural orifice transluminal endoscopic surgery vs laparoscopic hysterectomy. Am J Obstet Gynecol Glob Rep 2024.*

#### Required blood transfusions

**Figure 12:**
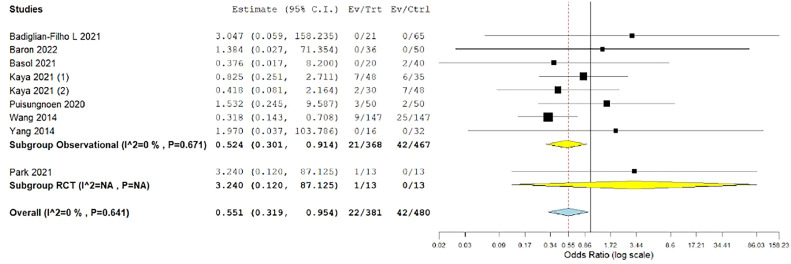
Meta-analysis of the rate of required blood transfusions *CI*, confidence interval; *RCT*, randomized controlled trial.

The requirement for blood transfusion was evaluated in 9 studies.^27^, ^28^, ^29^^,^^31^^,^^32^^,^^35^, ^36^, ^37^^,^^39^ The estimated OR favored the vNOTES group significantly (OR, 0.551; 0.319–0.954; *P*=.033). The data were homogeneous (*P*=.641; I²=0%) (Figure 12).

*Marchand. Vaginal natural orifice transluminal endoscopic surgery vs laparoscopic hysterectomy. Am J Obstet Gynecol Glob Rep 2024.*

## Discussion

### Initial context

The choice of minimally invasive surgical technique for hysterectomy for benign disease is a constant question of controversy and debate in gynecology.^41^ When comparing vNOTES with conventional laparoscopic techniques that use the abdominal wall as access, some benefits of vNOTES include decreased postoperative pain, accelerated postoperative recovery, and enhanced cosmesis.^9^ The method used to conduct a hysterectomy for an underlying condition depends on a number of variables, including the patient's preferences, the surgeon's training and preferences, the uterus size, accessibility, and extrauterine disease.

### Principal findings

In this study, we have found that the operative time and the length of hospital stay were shorter for the vNOTES techniques than for the laparoscopic techniques. In addition, the vNOTES technique was accompanied by a significantly lower VAS score on the first day after surgery. Regarding the postoperative complication rate, our analysis showed that the vNOTES technique was accompanied by a lower incidence of postoperative complications than the laparoscopic techniques. In addition, patients who underwent a hysterectomy in which the vNOTES technique was used needed blood transfusions less often than patients who underwent a hysterectomy in which laparoscopic techniques were used. In contrast, both procedures had no substantial variations in terms of estimated blood loss, the decrease in hemoglobin levels, the required analgesic doses, the conversion rate to another surgical technique, or intraoperative complications.

### Comparison with existing literature

A recent systematic review on this topic was published by Chaccour et al^6^ in May 2023 and included 7 articles. Its findings were consistent with ours in that they found that the vNOTES technique was accompanied by less operative time, less recovery time, and a lower incidence of postoperative pain and postoperative complications. Their analysis revealed that both techniques were similar in terms of the estimated blood loss, decrease in hemoglobin levels, and the incidence of perioperative complications.

In 2021, Michener et al^42^ conducted a meta-analysis that compared the vNOTES technique, laparaendoxcopic single site surgery (LESS), and the conventional laparoscopic hysterectomy for the management of malignant and benign gynecologic lesions. They found that both the vNOTES technique and LESS were superior to the conventional laparoscopic hysterectomy because these techniques were associated with less operative time, less recovery time, and a lower incidence of postoperative pain and complications. These findings are similar to ours.

In 2016, Baekelandt et al^9^ conducted a systematic review and meta-analysis that included only 2 retrospective studies. Similar to our results, they found that the vNOTES technique was accompanied by less operative time and less hospital stay time with an MD of −22.04 minutes and −0.42 days, respectively. In addition, they reported that the analgesic dose and the incidence of perioperative complications were the same in both cohorts without significant variations. Housmans et al^43^ performed an update for Baekelandt et al^9^ and included 6 studies that compared the vNOTES technique with a laparoscopic hysterectomy for managing benign lesions. Their analysis revealed the same results as our study and that of Baekelandt et al;^9^ they concluded that the values for procedure time, hospital stay, and estimated blood loss were all significantly reduced in vNOTES. There was no apparent variance in intraoperative and postoperative complications, readmission, pain scores at 24 hours following surgery, or hemoglobin changes.

Yang et al^39^ investigated the surgical results of a vNOTES hysterectomy and a single-port laparoscopy-assisted vaginal hysterectomy (SP-LAVH). They discovered no differences in the perioperative outcomes, such as estimated blood loss, the drop in hemoglobin, the number of analgesics used, febrile complications, and postoperative VAS pain scores. However, when compared with SP-LAVH, NOTES-assisted vaginal hysterectomy had shorter operating times, decreased days of postoperative hospital stay, and better cosmetic results.

The surgical results of a LAVH and those of a vNOTES hysterectomy were compared in another study of a similar nature by Wang et al.^37^ In comparison with LAVH, they discovered that a hysterectomy using vNOTES had a shorter operating time, less estimated blood loss, less need for blood transfusions, and shorter postoperative stays. The overall incidence of surgical complications was comparable across the 2 cohorts, but the total hospital costs were greater in the vNOTES cohort. Kim et al^33^ evaluated the postoperative results of NOTES-assisted vaginal hysterectomy and conventional LAVH for benign uterine illness from 2012 to 2015. They noticed that although the LAVH required less time during surgery, the NOTES-assisted vaginal hysterectomy led to a smaller decrease in hemoglobin levels. There were no variations between the cohorts in terms of intraoperative complications, additional procedures, changing the surgical technique, hospital stay, postoperative hemorrhage, and postoperative fever.

Park et al^35^ found that when compared with the total laparoscopic hysterectomy (TLH) cohort, the vNOTES hysterectomy cohort surgery required considerably less time (79.56±32.54 minutes vs 120.67±38.35 minutes; *P*<.001). In addition, the vNOTES hysterectomy cohort postoperative hospital stays (44±16.47 hours) were significantly shorter than that of the TLH cohort (57.86±21.31 hours).

### Strengths and limitations

The major strength of our study is that this was a large meta-analysis on this topic, and a relative abundance of publications on this topic has given us the opportunity to include more than double the number of studies than included in the largest previous analysis on this subject. A limitation of our study is that most of the included studies were retrospective studies, which may lead to a risk of measurement bias. In addition, significant heterogeneity was seen in many of our outcomes, and we were unable to solve this heterogeneity in many cases with a subgroup analysis. This may be attributed to the difference in the study designs of the included studies and the different laparoscopic techniques that were used in these studies. Lastly, the major limitation of our study was the necessity to combine laparoscopic techniques, which could introduce bias because individual techniques may have higher efficacy or safety. Because the number of available studies was too small for subgroup analyses of individual techniques, we cannot say with certainty that 1 specific technique, (LESS, for example,) is on its own superior or inferior to vNOTES.

### Conclusions and implications

The vNOTES hysterectomy is a unique method of minimally invasive hysterectomy. Furthermore, we discovered that the vNOTES approach was comparable with a laparoscopy for each measurement we assessed and superior in operative time, length of hospitalization, pain scores on postoperative day 1, postoperative complications, and the rate of blood transfusions. We hope to see additional research on this subject in the future, especially prospective, multicenter randomized trials with additional outcomes on financial burdens and the long-term health included.

## CRediT authorship contribution statement

**Greg J. Marchand:** Conceptualization, Data curation. **Ahmed Taher Masoud:** Data curation, Formal analysis, Investigation, Methodology. **Hollie Ulibarri:** Data curation, Validation, Writing – original draft. **Amanda Arroyo:** Data curation, Formal analysis, Investigation, Writing – original draft. **Carmen Moir:** Data curation, Formal analysis, Writing – original draft. **Madison Blanco:** Data curation, Supervision, Validation, Writing – original draft. **Daniela Gonzalez Herrera:** Data curation, Formal analysis, Visualization, Writing – review & editing. **Brooke Hamilton:** Data curation, Formal analysis, Methodology, Writing – original draft. **Kate Ruffley:** Data curation, Formal analysis, Writing – review & editing. **Mary Petersen:** Data curation, Formal analysis, Writing – review & editing. **Sarena Fernandez:** . **Ali Azadi:** Conceptualization, Formal analysis, Supervision.
